# Risk incidence of fractures and injuries: a multicenter video-EEG study of 626 generalized convulsive seizures

**DOI:** 10.1007/s00415-020-10065-5

**Published:** 2020-07-10

**Authors:** Katharina Frey, Johann Philipp Zöllner, Susanne Knake, Yulia Oganian, Lara Kay, Katharina Mahr, Fee Keil, Laurent M. Willems, Katja Menzler, Sebastian Bauer, Susanne Schubert-Bast, Felix Rosenow, Adam Strzelczyk

**Affiliations:** 1 Center of Neurology and Neurosurgery, Epilepsy Center Frankfurt Rhine-Main, Goethe-University Frankfurt, Schleusenweg 2-16 (Haus 95), 60528 Frankfurt am Main, Germany; 2 LOEWE Center for Personalized and Translational Epilepsy Research (CePTER), Goethe-University Frankfurt, Frankfurt am Main, Germany; 3 Epilepsy Center Hessen and Department of Neurology, Philipps-University Marburg, Marburg (Lahn), Germany; 4 Department of Neurological Surgery, University of California, San Francisco, CA USA; 5 Department of Neuroradiology, Goethe-University Frankfurt, Frankfurt am Main, Germany; 6 Department of Neuropediatrics, Goethe-University Frankfurt, Frankfurt am Main, Germany

**Keywords:** Epilepsy, Seizure, Morbidity, Fracture, Shoulder luxation

## Abstract

**Objective:**

To evaluate the incidence and risk factors of generalized convulsive seizure (GCS)-related fractures and injuries during video-EEG monitoring.

**Methods:**

We analyzed all GCSs in patients undergoing video-EEG-monitoring between 2007 and 2019 at epilepsy centers in Frankfurt and Marburg in relation to injuries, falls and accidents associated with GCSs. Data were gathered using video material, EEG material, and a standardized reporting form.

**Results:**

A total of 626 GCSs from 411 patients (mean age: 33.6 years; range 3–74 years; 45.0% female) were analyzed. Severe adverse events (SAEs) such as fractures, joint luxation, corneal erosion, and teeth loosening were observed in 13 patients resulting in a risk of 2.1% per GCS (95% CI 1.2–3.4%) and 3.2% per patient (95% CI 1.8–5.2%). Except for a nasal fracture due to a fall onto the face, no SAEs were caused by falls, and all occurred in patients lying in bed without evidence of external trauma. In seven patients, vertebral body compression fractures were confirmed by imaging. This resulted in a risk of 1.1% per GCS (95% CI 0.5–2.2%) and 1.7% per patient (95% CI 0.8–3.3%). These fractures occurred within the tonic phase of a GCS and were accompanied by a characteristic cracking noise. All affected patients reported back pain spontaneously, and an increase in pain on percussion of the affected spine section.

**Conclusions:**

GCSs are associated with a substantial risk of fractures and shoulder dislocations that are not associated with falls. GCSs accompanied by audible cracking, and resulting in back pain, should prompt clinical and imaging evaluations.

## Introduction

Video-EEG monitoring is required in many cases to differentiate between epilepsy and other possible diagnoses, to determine epilepsy type, and is indispensable for presurgical evaluation of epilepsy. Its diagnostic utility has been demonstrated in several studies [1–5].

In parallel to the increasing use of video-EEG monitoring, questions about the safety of the procedure itself have arisen [6–8]. Epileptic seizures, especially generalized convulsive seizures (GCSs; focal to bilateral tonic–clonic seizures and generalized tonic–clonic seizures of generalized or unknown onset [9, 10]) are a common cause of accidents and injuries due to the seizure itself or to concomitant factors such as seizure-induced falls or reduced awareness [11]. As seizure occurrence during video-EEG monitoring is, in part, artificially provoked by the discontinuation of anti-seizure drugs (ASDs) or by sleep deprivation, informed patient consent about related risks must be detailed and supported by reliable and relevant data.

The aim of this study was to analyze a large number of GCSs to be able to estimate the associated risk incidence for injuries, and to identify potential risk factors for adverse events related to GCSs.

## Methods

In this retrospective multicenter study, we evaluated the medical records of all patients who underwent video-EEG monitoring at epilepsy centers in Frankfurt and Marburg between January 2007 and June 2019. The study was approved by the local ethics committees, and we adhered to the Strengthening the Reporting of Observational Studies in Epidemiology (STROBE) guidelines [12].

The video-EEG monitoring was either performed for presurgical evaluations [13] in drug-refractory patients, or for the classification of epilepsy type. Withdrawal of ASDs or sleep deprivation were used to record a sufficient number of seizures. Classifications of seizure type, epilepsy type, and syndromes were adopted based on the latest definitions proposed by the International League Against Epilepsy (ILAE), and the semiological seizure classification [9, 14, 15]. In general, scalp electrodes using the 10–20 and 10–10 international systems were used for EEG-recordings, and in some patients with clinical indications, sphenoidal electrodes or intracranial electrodes were applied. The relevant quality guidelines of the working group for presurgical epilepsy diagnosis and operative epilepsy treatment were followed [13].

Inclusion criteria were the diagnosis of epilepsy, and at least one video-EEG record of a GCS. The abbreviation GCS refers to focal to bilateral tonic clonic seizures and generalized tonic–clonic seizures of generalized or unknown onset [9]. We assessed the incidence of all adverse events, and defined serious adverse events (SAEs) as any untoward medical occurrence that caused prolongation of an existing hospitalization, resulted in persistent or significant disability or incapacity, or required intervention to prevent permanent impairment or damage.

Using a standardized form, information was gathered concerning etiology, duration of epilepsy, habitual seizure frequency, current ASD treatment and previously used ASDs. Anonymized datasets generated during this study are available from the corresponding author upon reasonable request.

All GCSs were analyzed based on the corresponding video recordings, EEG data, and documentation by attending physicians and the nursing staff. Patients were excluded from the study if video records were not available for entire seizure durations. GCS onset was often characterized by versive head or body movement, or by vocalization. Referring to the classification of Theodore et al. [16], GCSs were categorized by different phase names such as generalization, clonic jerking, tonic phase, jittery tremulous, clonic phase, and by assessing the timespan of each phase.

Audio recordings from patient rooms during GCSs were manually annotated for fracture events and evaluated together with the corresponding EEG and video recordings. To characterize the audio characteristics of fracture events, we calculated spectrograms in the range of 0.01–20 kHz. Then, the average sound intensities below 1 Hz, and in the range of 3–15 Hz, were calculated as average of intensities for single spectrogram bands in each range, respectively. These two ranges were chosen to contrast the frequency content of fracture events in the range that corresponds to the main pitch content of human speech (< 1 Hz), and a high-frequency range that is typically only weak in human speech. Finally, audio temporal dynamics during fracture events were characterized by taking the first temporal derivative of the overall sound intensity in the range of 0.01–20 Hz.

Patients who sustained SAEs during monitored GCSs were compared to a matched patient group without SAEs to evaluate potential risk factors. Each patient with a SAE was matched to five patients without SAEs and their first GCS, matching was based on the adjacent admission to video-EEG-monitoring.

Data were analyzed using SPSS Statistics version 26 (IBM Corp., Armonk, NY, USA). Comparisons between groups were performed using the Wilcoxon–Mann–Whitney test for nonparametric values. Pearson’s chi-squared tests were used to assess the distributions of clinical characteristics between groups. The 95% confidence intervals (95% CIs) for the risks to sustain per-patient, and per GCS, SAEs were calculated by the Jeffreys method. All *p *values were two-sided and regarded as statistically significant at less than 0.05.

## Results

### Demographic and clinical characteristics

Generalize convulsive seizures were recorded in 411 patients [mean age: 33.6 years; median 31.0 years; range 3–74 years; *n* = 185 (45.0%) female] during the study period. Patients suffered for a mean of 15.5 years (SD 13.3; median 11.0 years; range 0.2–63 years) from either focal epilepsy (*n* = 341, 83.0%), genetic generalized epilepsy (*n* = 44, 10.7%) or another (or unknown) epilepsy syndrome (*n* = 26, 6.3%). Patients were treated with a mean of 2.1 ASDs, with a previous-treatment mean of 2.6 ASDs; for details refer to Table 1. At admission, 65.9% (*n* = 271/411) of patients reported having suffered from a GCS within the last 12 months, 29.7% (*n* = 122) denied a GCS during that time period, and 4.4% (*n* = 18) were unsure.

**Table 1:** Demographic and clinical patient characteristics

Patients (*n* = 411)	% (*n*)
Age in years	Mean 33.6 ± 13.7 Median 31.0 Range 3–74
Age at epilepsy onset	Mean 17.5 ± 12.5 Median 15.0 Range 0.3–67
Epilepsy duration in years	Mean 15.5 ± 13.3 Median 11.0 Range 0.2–63
Epilepsy syndrome	% (*n*)
Focal epilepsy	83.0 (341)
Idiopathic (genetic) generalized epilepsy	10.7 (44)
Other or unknown syndrome	6.3 (26)
Drug-refractory epilepsy	88.3 (363)
Overall seizure frequency prior to admission	
At least one seizure per day	10.5 (43)
At least once a week	29.7 (121)
At least once a month in	38.5 (157)
Less than once a month or unknown	21.9 (90)
Antiseizure drugs	Mean 2.1 ± 0.8 Median 2.0 Range 0–5
Number of antiseizure drugs used in the past^*^	Mean 2.6 ± 2.6 Median 2.0 Range 0–16
Current antiseizure drugs	% (*n*)
Levetiracetam	55.2 (227)
Lamotrigine	41.6 (171)
Lacosamide	20.7 (85)
Oxcarbazepine	18.5 (76)
Valproate	14.4 (59)
Zonisamide	10.9 (45)
Carbamazepine	9.0 (37)
Brivaracetam	7.1 (29)
Topiramate	6.6 (27)
Pregabalin	4.1 (17)
Clobazam	2.9 (12)
Perampanel	2.7 (11)
Eslicarbazepine	2.7 (11)
Other	5.1 (21)

*current antiseizure drugs not included

The mean duration of video-EEG recordings was 150 h (SD 66.0; median 120.0 h; range 24–480 h) and a total of 2,534 days of video-EEG monitoring were evaluated. In the majority (*n* = 387; 95.3%) of patients, ASDs were reduced or discontinued during video-EEG monitoring, while in 19 patients (4.7%) medication treatment was unchanged.

In total, 626 GCSs were analyzed, with a mean number of 1.6 (median 1.0; range 1–8) per patient. A single GCS was recorded in 246 patients (59.9%), while 112 patients (27.3%) suffered two, 33 (8.0%) suffered three, and 20 (4.9%) suffered four or more GCSs.

### Adverse events

Within the 12-year observation period, SAEs such as fractures, joint luxation, corneal erosion, or teeth loosening occurred during 13 GCSs (2.1% per GCS; 95% CI 1.2–3.4%) in 13 patients (3.2% of the total patient cohort; 95% CI 1.8–5.2%). Except for a nasal fracture due to a fall onto the face, no SAE was caused by a fall, and all occurred in patients lying in bed without any evidence of external trauma. We observed further adverse events (tongue biting, lip biting, lacerations, bruises, and nose bleeds), rated as minor injuries, in 49 patients (11.9%) and 55 GCSs (8.8% per GCS).

**Fig. 1:**
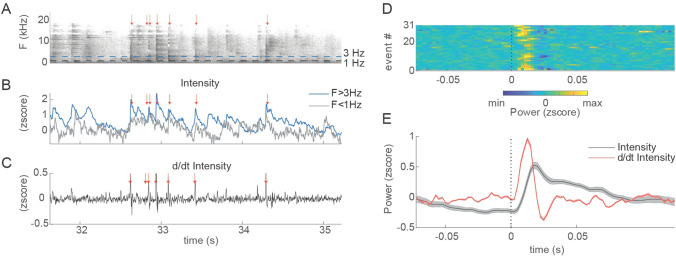
Digital analysis of vertebral-body fracture sounds. **a** Spectrogram of click sounds accompanying fracture events in one patient example. Red arrows mark time points of single-fracture clicks. Horizontal lines indicate boundaries of the frequency ranges, for which average sound intensities are plotted in **b**. **b** Average sound intensities in the low-frequency range (gray, 1 Hz) and high-frequency range (blue, > 3 Hz) for the same fracture events in **a**. **c** Temporal derivatives of overall sound intensities for the events depicted in **a** and **b**. Fracture events were characterized by rapid, transient increases in sound intensity across the entire frequency range. **d** Average power across the entire frequency range for *n* = 31 detected fracture-click sounds, aligned by time-of-sound. **e** Click events continued for approximately 25 ms

Among the SAEs, seven patients sustained vertebral-body compression fractures (1.7% [95 CI 0.8–3.3%] of the total patient cohort; 1.1% per GCS [95 CI 0.5–2.2%]) four of which affected the thoracic spine, and three of which affected the lumbar spine. Details of these injuries, patient characteristics, and SAE outcomes are presented in Table 2. Times of vertebral-body fractures could be identified in five of the seizures by identification of a characteristic broadband sound of breaking bone. This observation was supported by digital analysis of audio recordings (Fig. 1). Figure 1a–c shows an example spectrogram (a), intensity levels (b), and temporal modulation of intensity (c) for the sound of fracture in one patient. Perceptually, fractures sounded like high-frequency clicks, and typically occurred in rapid, irregular series of 2–10 clicks per second. In the spectral domain, fracture clicks had broad frequency content, with high intensities present in the entire range up to 20 kHz (Fig. 1a, b). Temporally, fracture clicks were characterized by a rapid, transient increase in intensity (Fig. 1c). Across five patients, we identified 33 fracture-click sounds (Fig. 1d). On average, all events had a similar temporal extent of approximately 25 ms (Fig. 1e).

**Table 2:** Clinical and seizure characteristics of, and diagnostic and therapeutic procedures in, 13 patients with severe adverse events due to generalized convulsive seizures (GCSs)

Patient #	Age (years)	Epilepsy duration (years)	Sex	Epilepsy syndrome	Total GCS	Seizure onset	Seizure EEG lateralization	Position during GCS	Comorbidities	Injury	Diagnostics and treatment
1	17	1	Male	FE (FLE)	2	Sleep	Left	Supine, in bed, no fall	Hyponatremia	Shoulder luxation	X-ray, splinting
2	19	5	Male	FE (TLE)	1	Sleep	Left	Right lateral and supine, in bed, no fall	Nicotine abuse	Fracture vertebral bodies (Th3-4)	CT, conservative treatment
3	20	16	Male	FE (OLE)	2	Sleep	Right	Left lateral, in bed, no fall	None	Corneal erosion	Ophthalmological examination, conservative treatment
4	22	2	Male	FE (TLE)	2	Sleep	Right	Supine, in bed, no fall	Von Willebrandt disease	Shoulder luxation and scapula fracture	X-ray, arthroscopy, fracture reposition
5	22	17	Male	GGE	1	Awake	Bilateral	Supine, in bed, no fall		Shoulder subluxation	X-ray, splinting
6	26	16	Male	FE (FLE)	1	Awake	Left	Supine, in bed, no fall	Previous vertebral fracture and of left zygomatic bone and orbita due to GCS	Fracture vertebral bodies (Th5-8)	MRI, conservative treatment
7	32	15	Male	FE (FLE)	2	Sleep	Left	Left lateral and prone, in bed, no fall	Nicotine abuse	Fracture vertebral bodies (Th3-8)	MRI, conservative treatment
8	32	16	Male	GGE	1	Awake	Bilateral	Supine, in bed, no fall	None	Fracture vertebral body (L5)	X-ray, CT, MRI, conservative treatment
9	35	7	Male	FE (TLE)	1	Sleep	Left	Left lateral and supine, in bed, no fall	None	Fracture vertebral bodies (Th12-L1)	CT, dorsal stabilization
10	36	21	Male	FE (FLE)	1	Awake	Right	Supine, in bed, no fall	Sinus bradycardia	Fracture and loosening of the teeth	Splinting
11	45		Female	FE (FLE)	3	Awake	Right	Sitting in chair and fell to prone position	None	Fractur of the nasal bone	Cranial CT, conservative treatment
12	66	63	Female	FE (FLE)	1	Sleep	Left	Right lateral, in bed, no fall	None	Fracture vertebral body (L1)	CT, conservative treatment
13	69	58	Female	FE (TLE)	2	Sleep	Left	Left lateral, in bed, no fall	Arterial hypertension	Fracture vertebral bodies (Th4 & Th9)	X-ray, CT, kyphoplasty
	Ø 33.9	Ø 19.8	23% female 77% male	85% FE 15% GGE	Ø 1.5	38% awake 62% sleep	31% right 54% left 15% bilateral	92% in bed, no fall 8% with fall			

*FE* focal epilepsy, *FLE* frontal lobe epilepsy, *TLE* temporal lobe epilepsy, *OLE* occipital lobe epilepsy, *GGE* genetic generalized epilepsy

**Fig. 2:**
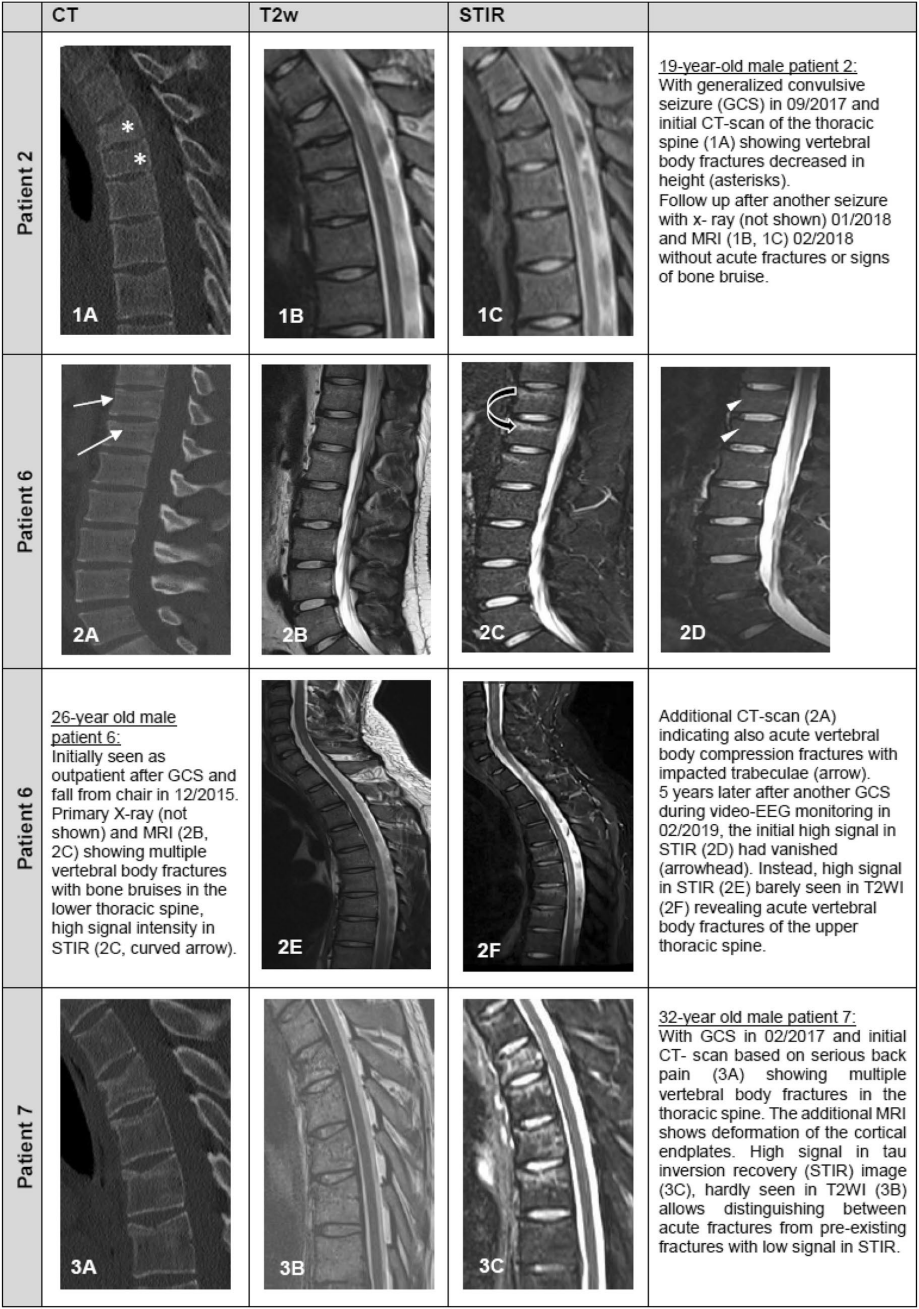
Imaging of spinal injuries

These fractures occurred within the tonic phase of GCSs. Following a GCS, patients reported spontaneous back pain, and increased pain on percussion of the affected spine area. All patients underwent diagnostic imaging (Fig. 2), five of the fractures were treated conservatively, and two fractures required surgical intervention.

### Risk factors for severe adverse events

**Fig. 3:**
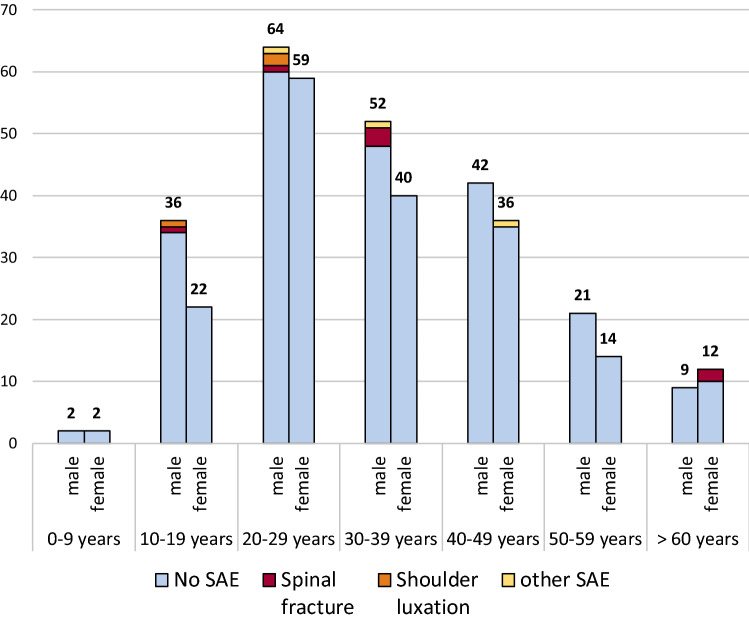
Age and gender distribution of patients, and severe adverse events (SAEs) due to generalized convulsive seizures

The matched groups, with and without SAEs, were well-matched for age (*p* = 0.972), but there was a trend towards male gender (*p* = 0.083) in the SAE group. Figure 3 shows the distribution of SAEs according to age and gender. Female patients with SAEs (mean age 60 years, SD 13.1) were significantly older than female patients without SAEs (mean age: 34.5 years, SD 16.3, *p* = 0.02), while the average age of males with SAEs (mean age: 26.1 years, SD 7.1) was lower than the average age of controls (mean age: 33.5 years, SD 17.1), but this difference did not reach statistical significance (*p* = 0.35). Patients with SAEs had longer video-EEG-monitoring than controls, but we did not detect any other differences among patients, video-EEGs, or seizure characteristics of GCSs (for details, refer to Table 3).

**Table 3:** Clinical and seizure characteristics in patients with severe adverse events (SAEs) and matched controls without SAEs

	Patients with SAE *n* = 13	Matched controls without SAE *n* = 65	*p* value
Patient characteristics			
Age in years	33.9 ± 16.9	34.0 ± 16.6	0.972
Gender	3 f/10 m	32 f/33 m	0.083
Epilepsy duration in years	19.7 ± 20.1	13.1 ± 12.1	0.348
Age at onset of epilepsy in years	13.3 ± 7.2	20.2 ± 17.1	0.293
Epilepsy syndrome			
Focal epilepsy	10 (76.9%)	51 (78.5%)	0.219
Genetic generalized epilepsy	2 (15.4%)	11 (16.9%)	
Other or unknown syndromes	1 (7.7%)	3 (4.6%)	
Drug-refractory epilepsy	11 (84.6%)	54 (83.1%)	0.89
Antiseizure drugs	2.2 ± 0.8	2.2 ± 0.8	0.874
Failed antiseizure drugs in the past	2.4 ± 2.4	2.6 ± 2.9	0.949
GCS within last 12 months			
Yes	11 (84.6%)	44 (67.7%)	0.221
No or unsure	2 (15.4%)	21 (32.3%)	
Video-EEG-Monitoring characteristics			
Duration of video-EEG in hours	184.6 ± 60.7	147.4 ± 61.4	0.044
Reduction of ASM	13 (100%)	64 (98.5%)	0.652
Total number of seizures	2.6 ± 3.0	4.4 ± 5.7	0.111
Total number of GCS	1.5 ± 0.7	1.7 ± 1.1	0.869
Seizure characterictics			
Duration of GCS in seconds	58.4 ± 11.8	66.7 ± 19.7	0.119
Duration of tonic phase in seconds	13.0 ± 6.7	10.2 ± 5.9	0.133

*ASM* anti-seizure medication; *GCS* generalized convulsive seizure

## Discussion

This study analyzed a large number of 626 consecutive GCSs and estimated the associated risk of SAEs at 2.1% per GCS and 3.2% per patient in whom GCS were recorded. During the 12-year observation period, seven patients sustained vertebral-body fractures resulting in a risk of 1.1% per GCS or 1.7% per recorded patient. Remarkably, none of the vertebral-body fractures was associated with a fall, and they occurred spontaneously during the tonic phase of GCSs. Clinicians should ask all patients who recently had a GCS about back or shoulder pain, pay attention to patients reporting these symptoms spontaneously following GCSs, and proceed with radiological examinations to prove, or rule out, shoulder and vertebral injuries or fractures. Our observation of a characteristic sound of vertebral-body breaking might add to clinical suspicions, and video-EEG data should be screened for that clinical sign. Audio-based seizure detection devices are under development [17] and a future application might be to search the audio recordings for the sound of vertebral-body breaking. In summary, the present data allows patients to be informed of the risks associated with GCSs during video-EEG monitoring in more detail.

Our findings are in line with other studies reporting increased risk of injuries and fractures in epilepsy patients compared to control patients [18–22]. Grzonka et al. performed a systematic review of bone fractures from GCSs and status epilepticus, including 39 studies and case reports [23]. They concluded that among all reported fractures, bilateral posterior fracture‐dislocations of the shoulders (33%), thoracic and lumbar vertebral compression fractures (29%), skull and jaw fractures (8%), and bilateral femoral neck fractures (6%) were the most common fracture locations [23]. Vertebral compression fractures were reported in eight (5.2%) out of 153 cases for thoracic, and in six (1.2%) out of 511 cases for lumbar locations. A large study of 2,800 patients admitted to hospital with a diagnosis of seizure showed fractures in 30 (1.1%) of them, while in 7 patients (0.3%), fractures were a consequence of seizure alone, without direct trauma [22].

Studies on adverse events during video-EEG monitoring units report lower numbers for fractures and injuries not related to falls. Dobesberger et al. analysed 596 consecutive video-EEG sessions, and reported 53 adverse events in 9% (44/507) of patients (definitive diagnoses of epilepsy in 400 of these patients) within a six-year period. Injuries were reported in 15 patients (3%), with minor injuries in 11 patients (bruises, abrasions, and lacerations), while two falls resulted in non-dislocated fractures of nasal bones, and one patient sustained a severe head injury (acute epidural hematoma) after a fall during a GCS. Two patients (a 37-year-old male and 73-year-old female) had compression fractures of lumbar vertebrae verified by spine computed tomography, and both presented with back pain after GCSs [7]. These authors implemented personalized safety measures to reduce adverse events during video-EEG monitoring and showed in a follow-up study that injuries decreased from 3 to 2% [6]. In their follow-up cohort, no vertebral fractures were reported. Strategies for fall prevention and bathroom safety were also implemented in other epilepsy-monitoring units, and resulted in a reduction in fall frequency and associated injuries [24]; however, there are no specific strategies available to prevent GCS-related fractures not associated with falls. Only the prevention of GCS can reduce the probability of associated fractures; however, this might interfere with recording enough seizures for presurgical evaluation.

Sauro et al. studied 396 consecutive patients with 101 recorded GCSs, and the most common adverse events were seizure-related injuries that were not related to falls in nine patients (2.3%), but the details of the injuries were not specified [25]. Ley et al. examined 175 patient cases with 195 recorded GCSs, and observed one case of double-vertebral compression fracture, one spinal disc herniation, and one relapse in a case of chronic glenohumeral luxation [26]. Fahoum et al. analyzed 524 consecutive admissions, and observed one nasal fracture due to a seizure-related fall [27]. At the other end of the scale, several studies reported no fractures during video-EEG monitoring. Craciun et al. studied 976 patients admitted for video-EEG monitoring and reported falls in 19 patients (1.9%), but none of them resulted in injury. Overall 177 GCSs were recorded [28]. Recently Cox et al. examined 1062 admissions, comprising 1518 video-EEG monitoring days, and reported no fractures. In ten patients, falls were reported (1%), but with no, or only minor, injuries [8]. In their cohort; however, seizures were only recorded in 256 patients out of 1062 admissions [8], so the reported risk of injuries and adverse events in video-EEG monitoring units will depend heavily on the number of patients in whom seizures, specifically GCSs, were recorded as these carry the highest risk for fractures and other injuries. However, the number of recorded GCSs is not provided in the majority of studies that focus on safety of video-EEG monitoring, making comparisons with our study difficult.

Recognition of fractures can be challenging, as symptoms may be misinterpreted as muscle pain, or as emerging rhabdomyolysis after a seizure or status epilepticus, and studies about injuries related to GCSs may be affected both by underreporting and by lack of awareness [23, 29]. Grzonka et al. suggested a three‐step screening procedure to identify fractures related to seizures. First, patients should be asked about postictal musculoskeletal pain, especially in their joints, back, and extremities. Second, clinical examinations should look for the presence of fractures by palpation of specific risk locations, and check for deformities, limited joint mobility, and bruising. Third, imaging of areas under suspicion should be performed [23]. Such recommendations mirror our own clinical approach, and may have resulted in the substantial number of vertebral compression fractures that were uncovered by computed tomography and magnetic resonance imaging. The latter allowed a delineation of acute and older fractures by use of short tau inversion imaging (STIR) to demonstrate edema and bone bruising (Fig. 2) [30].

We found a correlation between injuries and older age in females, a finding that might be attributed to a higher prevalence of osteoporosis in older women [31, 32]. However, epilepsy patients in general have a high risk of developing bone disease, caused by ASD intake as well as other factors [33, 34]. Accordingly, seizure severity, duration of epilepsy, the use of ASDs known to decrease bone mineral-density, and a family history of fractures have all been identified as risk factors for injuries [23].

### Strengths and limitations

The limitations of our analysis were its retrospective design, the artificial setting during video-EEG monitoring, reduction of ASDs, and recording and evaluating patients in hospital settings rather than in patients' home and work environments. The strengths of our study include the controlled conditions of continuous video and EEG monitoring, allowing us to detect seizure phases and the cracking noises associated with bone fractures, and the large number of patints with GCSs.

## Conclusion

Our results show that GCSs are associated with a substantial risk of fractures, or shoulder dislocations, not associated with falls. This finding is important both in the context of video-EEG monitoring, where patients should be informed about the risk incidence of injuries, and in the broader context of inpatient and outpatient treatments, where physicians should ask about and look for signs and symptoms of injuries after GCSs. We suggest that clinical practice guidelines recommend standardized screening for shoulder dislocations and bone fractures in patients after GCSs, with or without associated falls.

Before discontinuing ASDs during video-EEG monitoring, a risk–benefit analysis should be carried out together with the patient to discuss the benefits of presurgical evaluation, or the benefits of confirming the diagnosis of epilepsy and the resulting therapeutic consequences in both patient groups. This should particularly apply to patients who have not recently experienced GCS under therapy with ASDs, and who have known risk factors for fractures such as osteoporosis, previous fractures or family history of fractures.

## References

[1] Cascino GD (2002). Clinical indications and diagnostic yield of video-electroencephalographic monitoring in patients with seizures and spells. Mayo Clin Proc.

[2] Boon P, Michielsen G, Goossens L, Drieghe C, D'Have M, Buyle M, Vonck K, Naessens B, De Paemeleere F, Goethals I, Thiery E, Vandekerckhove T, De Reuck J (1999). Interictal and ictal video-EEG monitoring. Acta Neurol Belg.

[3] Chen LS, Mitchell WG, Horton EJ, Snead OC (1995). Clinical utility of video-EEG monitoring. Pediatr Neurol.

[4] Cho YW, Motamedi GK, Kim KT (2019). The clinical utility of non-invasive video-electroencephalographic monitoring has been diversifying. Neurol Sci.

[5] Rosenow F, Lüders H (2001). Presurgical evaluation of epilepsy. Brain.

[6] Dobesberger J, Höfler J, Leitinger M, Kuchukhidze G, Zimmermann G, Thomschewski A, Unterberger I, Walser G, Kalss G, Rohracher A, Neuray C, Kobulashvili T, Holler Y, Trinka E (2017). Personalized safety measures reduce the adverse event rate of long-term video EEG. Epilepsia Open.

[7] Dobesberger J, Walser G, Unterberger I, Seppi K, Kuchukhidze G, Larch J, Bauer G, Bodner T, Falkenstetter T, Ortler M, Luef G, Trinka E (2011). Video-EEG monitoring: safety and adverse events in 507 consecutive patients. Epilepsia.

[8] Cox F, Reus E, Widman G, Zwemmer J, Visser G (2020). Epilepsy monitoring units can be safe places; a prospective study in a large cohort. Epilepsy Behav.

[9] Fisher RS, Cross JH, French JA, Higurashi N, Hirsch E, Jansen FE, Lagae L, Moshe SL, Peltola J, Roulet Perez E, Scheffer IE, Zuberi SM (2017). Operational classification of seizure types by the international league against epilepsy: position paper of the ILAE commission for classification and terminology. Epilepsia.

[10] Rosenow F, Akamatsu N, Bast T, Bauer S, Baumgartner C, Benbadis S, Bermeo-Ovalle A, Beyenburg S, Bleasel A, Bozorgi A, Brazdil M, Carreno M, Delanty N, Devereaux M, Duncan J, Fernandez-Baca Vaca G, Francione S, Garcia Losarcos N, Ghanma L, Gil-Nagel A, Hamer H, Holthausen H, Omidi SJ, Kahane P, Kalamangalam G, Kanner A, Knake S, Kovac S, Krakow K, Kramer G, Kurlemann G, Lacuey N, Landazuri P, Lim SH, Londono LV, LoRusso G, Luders H, Mani J, Matsumoto R, Miller J, Noachtar S, O'Dwyer R, Palmini A, Park J, Reif PS, Remi J, Sakamoto AC, Schmitz B, Schubert-Bast S, Schuele S, Shahid A, Steinhoff B, Strzelczyk A, Szabo CA, Tandon N, Terada K, Toledo M, van Emde BW, Walker M, Widdess-Walsh P (2020). Could the 2017 ILAE and the four-dimensional epilepsy classifications be merged to a new “Integrated Epilepsy Classification”?. Seizure.

[11] Wirrell EC (2006). Epilepsy-related injuries. Epilepsia.

[12] von Elm E, Altman DG, Egger M, Pocock SJ, Gotzsche PC, Vandenbroucke JP (2007). Strobe initiative—The strengthening the reporting of observational studies in epidemiology (STROBE) statement: guidelines for reporting observational studies. Epidemiol.

[13] Rosenow F, Bast T, Czech T, Feucht M, Hans VH, Helmstaedter C, Huppertz HJ, Noachtar S, Oltmanns F, Polster T, Seeck M, Trinka E, Wagner K, Strzelczyk A (2016). Revised version of quality guidelines for presurgical epilepsy evaluation and surgical epilepsy therapy issued by the Austrian, German, and Swiss working group on presurgical epilepsy diagnosis and operative epilepsy treatment. Epilepsia.

[14] Scheffer IE, Berkovic S, Capovilla G, Connolly MB, French J, Guilhoto L, Hirsch E, Jain S, Mathern GW, Moshe SL, Nordli DR, Perucca E, Tomson T, Wiebe S, Zhang YH, Zuberi SM (2017). ILAE classification of the epilepsies: position paper of the ILAE commission for classification and terminology. Epilepsia.

[15] Lüders H, Vaca GF, Akamatsu N, Amina S, Arzimanoglou A, Baumgartner C, Benbadis SR, Bleasel A, Bermeo-Ovalle A, Bozorgi A, Carreno M, Devereaux M, Francione S, Losarcos NG, Hamer H, Holthausen H, Jamal-Omidi S, Kalamangalam G, Kanner AM, Knake S, Lacuey N, Lhatoo S, Lim SH, Londono LV, Mani J, Matsumoto R, Miller JP, Noachtar S, Palmini A, Park J, Rosenow F, Shahid A, Schuele S, Steinhoff BJ, Akos Szabo C, Tandon N, Terada K, Boas WVE, Widdess-Walsh P, Kahane P (2019). Classification of paroxysmal events and the four-dimensional epilepsy classification system. Epileptic Disord.

[16] Theodore WH, Porter RJ, Albert P, Kelley K, Bromfield E, Devinsky O, Sato S (1994). The secondarily generalized tonic-clonic seizure: a videotape analysis. Neurology.

[17] Shum J, Fogarty A, Dugan P, Holmes MG, Leeman-Markowski BA, Liu AA, Fisher RS, Friedman D (2020). Sounds of seizures. Seizure.

[18] Strzelczyk A, Griebel C, Lux W, Rosenow F, Reese JP (2017). The burden of severely drug-refractory epilepsy: a comparative longitudinal evaluation of mortality, morbidity, resource use, and cost using german health insurance data. Front Neurol.

[19] Willems LM, Watermann N, Richter S, Kay L, Hermsen AM, Knake S, Rosenow F, Strzelczyk A (2018). Incidence, risk factors and consequences of epilepsy-related injuries and accidents: a retrospective single center study. Front Neurol.

[20] Camfield C, Camfield P (2015). Injuries from seizures are a serious, persistent problem in childhood onset epilepsy: a population-based study. Seizure.

[21] Lawn ND, Bamlet WR, Radhakrishnan K, O'Brien PC, So EL (2004). Injuries due to seizures in persons with epilepsy: a population-based study. Neurology.

[22] Finelli PF, Cardi JK (1989). Seizure as a cause of fracture. Neurology.

[23] Grzonka P, Rybitschka A, De Marchis GM, Marsch S, Sutter R (2019). Bone fractures from generalized convulsive seizures and status epilepticus-a systematic review. Epilepsia.

[24] Spritzer SD, Riordan KC, Berry J, Corbett BM, Gerke JK, Hoerth MT, Crepeau AZ, Drazkowski JF, Sirven JI, Noe KH (2015). Fall prevention and bathroom safety in the epilepsy monitoring unit. Epilepsy Behav.

[25] Sauro KM, Macrodimitris S, Krassman C, Wiebe S, Pillay N, Federico P, Murphy W, Jette N, Epilepsy Monitoring Unit Quality Improvement T (2014). Quality indicators in an epilepsy monitoring unit. Epilepsy Behav.

[26] Ley M, Vivanco R, Massot A, Jimenez J, Roquer J, Rocamora R (2014). Safety study of long-term video-electroencephalogram monitoring. Neurologia (Barcelona, Spain).

[27] Fahoum F, Omer N, Kipervasser S, Bar-Adon T, Neufeld M (2016). Safety in the epilepsy monitoring unit: a retrospective study of 524 consecutive admissions. Epilepsy Behav.

[28] Craciun L, Alving J, Gardella E, Terney D, Meritam P, Cacic Hribljan M, Beniczky S (2017). Do patients need to stay in bed all day in the epilepsy monitoring unit? Safety data from a non-restrictive setting. Seizure.

[29] Sutter R, Dittrich T, Semmlack S, Ruegg S, Marsch S, Kaplan PW (2018). Acute systemic complications of convulsive status epilepticus-a systematic review. Crit Care Med.

[30] Winklhofer S, Thekkumthala-Sommer M, Schmidt D, Rufibach K, Werner CM, Wanner GA, Alkadhi H, Hodler J, Andreisek G (2013). Magnetic resonance imaging frequently changes classification of acute traumatic thoracolumbar spine injuries. Skeletal Radiol.

[31] Verboket RD, Sohling N, Marzi I, Paule E, Knake S, Rosenow F, Strzelczyk A, Willems LM (2019). Prevalence, risk factors and therapeutic aspects of injuries and accidents in women with epilepsy. Eur J Trauma Emerg Surg.

[32] Compston JE, McClung MR, Leslie WD (2019). Osteoporosis. Lancet.

[33] Diemar SS, Sejling AS, Eiken P, Andersen NB, Jorgensen NR (2019). An explorative literature review of the multifactorial causes of osteoporosis in epilepsy. Epilepsy Behav.

[34] Bauer S, Hofbauer LC, Rauner M, Strzelczyk A, Kellinghaus C, Hallmeyer-Elgner S, Oertel WH, Rosenow F (2013). Early detection of bone metabolism changes under different antiepileptic drugs (ED-BoM-AED)–a prospective multicenter study. Epilepsy Res.

